# Alcohol drinking among college students: college responsibility for personal troubles

**DOI:** 10.1186/1471-2458-13-615

**Published:** 2013-06-28

**Authors:** Vincent Lorant, Pablo Nicaise, Victoria Eugenia Soto, William d’Hoore

**Affiliations:** 1 Institute of Health and Society, Université catholique de Louvain, Clos Chapelle-Aux-Champs B1.30.15, B-1200 Brussels, Belgium; 2 Institute for Health and Society, Clos- chapelle aux champs 30 - B1.30.15.05, 1200 Brussels, Belgium

**Keywords:** Alcohol drinking, College students, Public health, Community health, Belgium

## Abstract

**Background:**

One young adult in two has entered university education in Western countries. Many of these young students will be exposed, during this transitional period, to substantial changes in living arrangements, socialisation groups, and social activities. This kind of transition is often associated with risky behaviour such as excessive alcohol consumption. So far, however, there is little evidence about the social determinants of alcohol consumption among college students. We set out to explore how college environmental factors shape college students' drinking behaviour.

**Methods:**

In May 2010 a web questionnaire was sent to all bachelor and master students registered with an important Belgian university; 7,015 students participated (participation = 39%). The survey looked at drinking behaviour, social involvement, college environmental factors, drinking norms, and positive drinking consequences.

**Results:**

On average each student had 1.7 drinks a day and 2.8 episodes of abusive drinking a month. We found that the more a student was exposed to college environmental factors, the greater the risk of heavy, frequent, and abusive drinking. Alcohol consumption increased for students living on campus, living in a dormitory with a higher number of room-mates, and having been in the University for a long spell. Most such environmental factors were explained by social involvement, such as participation to the student folklore, pre-partying, and normative expectations.

**Conclusions:**

Educational and college authorities need to acknowledge universities’ responsibility in relation to their students’ drinking behaviour and to commit themselves to support an environment of responsible drinking.

## Background

In 2007 one young adult in two has entered university education in Western countries and this proportion is likely to increase in the future [1]. Many of these young students will be exposed to substantial changes in living arrangements and social activities. This kind of transition is often associated with an increase in heavy and risky alcohol use [2].

Indeed, it is reckoned that college students are particularly exposed to alcohol during their college years. An international study of alcohol consumption among students found wide geographical variation in the prevalence of risky drinking behaviour, with more than 40% of students aged 17-30 having drunk heavily in the U.S.A. and in several European countries [3]. Risky drinking has also been found to be a common practice [4].

Risky alcohol consumption among young people is becoming a key public health priority because of its important health and educational consequences. Among those aged 15-29, alcohol accounts for more than 10% of the overall burden of disease and injury [5]. In addition to morbidity and mortality, alcohol has a significant important effect on student academic performance and on antisocial behaviour [6,7]. The case for alcohol could be weakened if adolescent drinking patterns became more mature in adulthood. However, a review of cohort studies shows that higher consumption in late adolescence continues into adulthood [8].

Risky alcohol consumption has first been approached from an individual perspective, with a strong emphasis on individual risk factors, such as gender, age, and psychological factors, and on drinking motives [3,9,10]. Adolescents often report drinking for motives such as social enhancement, enjoyment, image enhancement, or coping motives; thus, they may drink because of positive consequences that outweigh, at least in the short term, negative consequences [11-13].

International comparison, however, shows there is wide cross-country variation in the prevalence of risky drinking among college students [3]. Within the U.S.A., there is compelling evidence that college drinking varies dramatically between colleges [14]. This indicates that alcohol use may be sensitive to contextual factors. Alcohol use among college students occurs in specific social environments characterised by independent living, reduced parental control, increased social homogeneity, wide availability of alcohol-related social activities such as pre-partying [15] and student folklore (traditional, extra-curricular, and generally recreational activities managed by student organisations) [16]. The transition to the college environment brings about changes in adolescents’ adjustment to their social environment, which in turns influence alcohol use [2]. We thus need to better understand upstream factors that shape drinking at college. With some exceptions [10], there has, however, been little research into the college-related environmental risk factors affecting drinking by college students in Europe. Moreover, research needs to better understand the contribution of the college and university context to alcohol-drinking behaviour. The European university system and legal provisions related to alcohol consumption differ considerably from those in North America. Thus, research of the kind suggested might indicate opportunities for community health preventive interventions.

### Objectives

The study analyses alcohol consumption among college students from a community health perspective. We aim to understand how the college-related environment shapes students’ drinking behaviour. In particular, we assess the role of living arrangements, college social activities, and social norms in drinking patterns. We explore two questions: (1) does the college-related environment influence alcohol use? (2) How do social and normative factors contribute to these college influences on alcohol use?

## Methods

### Design and participants

This study is part of an important multi-method investigation into alcohol drinking among college students. It was carried out in a Belgian university with two main campuses, one in Louvain-La-Neuve, a town of 20,000 inhabitants, half of whom are students living in dormitories. The other campus, mainly devoted to health sciences, is located in Brussels, 30 km away from the main campus.

A web survey was carried out in May 2010. An e-mail invitation was sent to all bachelor and master students registered with the university (n = 18,137), with a link to a web-survey questionnaire. No financial or material incentive was provided. The students could request a copy of the final report, and 62% of the respondents made such request. The form included 31 questions related to socio-demographics, living arrangements, study programmes, involvement in student activities, alcohol use, injunctive and descriptive norms, and positive and negative consequences of alcohol use. On average, filling in the questionnaire took 12 minutes and very few break-offs were recorded. The study was approved for ethical issues by the Social and Student Affairs review board of the university on the 26^th^ March 2010.

After up to two reminders, 7,015 students (39%) participated, a rate well above the average web survey participation rate [17]. Compared with a face-to-face survey, our participation rate may look low. But for a web survey it is a very satisfactory result, as it corresponds to the median web-survey participation [18]. In addition, there is no evidence that online surveys with lower response rates produce biased estimates in higher education evaluation or surveys [19] or in surveys in general [20,21]. However, to assess the risk of bias we compared the distribution of our sample with the distribution of the population. Analysis of non-participants suggests that women were a bit more likely to participate (OR = 1.10) than men; no differences of participation regarding age or year of study were noted. There is thus little evidence of an important bias linked to factors associated with alcohol consumption.

### Measures

Alcohol consumption measures came from the Eurostat European Health Interview Survey schedule and from the European School Survey Project on Alcohol and Other Drugs (ESPAD) questionnaire [22]. We modelled the weekly average number of drinks in the last year, the monthly frequency of drinking, and the monthly frequency of abusive drinking (more than 6 drinks on one occasion [23]). A drink was defined as a glass of any alcoholic beverage (beer, wine, spirits, other), assuming that a standardised glass of beer, wine, or spirit contains a similar quantity of alcohol (from 10 to 13 g).

College environmental factors included curricular and extra-curricular features. The former consisted of the number of years the student had been studying and the study programme (a student class within a curriculum). The study programme factor was expected to capture the peer effect linked to the culture of alcohol consumption specific to faculties. The extra-curricular features were: living arrangements (living in a dormitory or with parents), living on the campus (yes or no), and the number of room-mates (0 for staying with parents).

Social involvement was measured by involvement in traditional student folklore, pre-partying, and being a student representative. Student folklore shares some similar features with the sororities and fraternities in the U.S.A. It has long played a traditional role in European university social life: traditional students’ organisations contribute to welcoming freshmen and to rites of passage; they organise parties and other recreational activities that may or may not involve drinking. Some student associations also manage student accommodation and sit on consultative bodies related to student social affairs. Involvement in this kind of traditional student folklore was measured by a score ranging from a low of 0 to a high of 3 according to rites of passage or positions of responsibility in student folklore. One point was given for each of the following: participation in hazing activities at the beginning of the academic year, after which one is labelled “baptisé”; participation in another traditional activity that upgrades the student’s prestige and allows him/her to wear a ritual cap called a “calotte”; and participation in the folklore organisation. The score was categorised in three groups (0 = none, 1 = medium, 2-3 = high). Finally, the university provides students with many curricular or extra-curricular social organisations. We asked the students whether they were members of any organisation of that kind.

Pre-partying was defined as the consumption of alcohol with friends while preparing to go out for the night. Pre-partying helps to improve sociability and conviviality, easing the discomfort associated with meeting new people at a party [15,24-26]. Students were requested to report their pre-partying frequency per month.

The normative factors included descriptive and injunctive norms. Alcohol injunctive norms were covered by a four-item questionnaire measuring approval by friends of four kinds of drinking behaviour: drinking every weekend, daily, after driving, and enough to be drunk [27]. Each item had a score ranging from 0 (strong disapproval) to 4 (strong approval). The overall sum of the four items ranged between 0 and 16 and measures “permissiveness”. Descriptive norms measure the perceived drinking behaviour of referent others and were assessed by the Drinking Norm Rating Forms, which ask the student to estimate the average daily number of drinks individuals of three different reference groups consume (students in general, same-sex students, and friends) [27]. According to social comparison theory proximal comparisons are more relevant than distal comparisons, so we expected that friends’ average consumption would have a greater influence than typical same-sex student consumption [28].

In order to also include experiential reporting, students’ positive drinking consequences were registered via the Positive Drinking Consequences Questionnaire, a 14-item scale [29]. This scale measures actual and past perceived positive consequences of alcohol use and differs from expectations. The scale mainly records consequences of drinking in terms of improved social interaction (11 items out of 14) such as social enhancement and stress reduction. This seemed relevant for young people in transition to adulthood and experiencing a dramatic change in their living conditions. We counted the number of times students reported a positive consequence of drinking over the last year.

### Data analysis

The analysis was in two stages, according to our two research questions. First, we investigated the role of socio-demographics and college environmental factors. Second, we added to the analysis social-involvement, normative, and experiential factors that might contribute to the influence of college environmental factors. However, cross-sectional analysis of drinking behaviour is vulnerable to selection bias: unobserved heterogeneity across individuals may explain why some vulnerable individuals self-select into an at-risk college environment, as predicted by the theory of increased heterogeneity [2]. We assessed this kind of bias by sensitivity analysis: we checked the robustness of the models by including age at first drink, a factor strongly linked to poor executive function and an individual risk factor for subsequent drinking and drug abuse [30]. Because number of drinks and frequency of abusive drinking are not normally distributed and because of over-dispersion (a minority may never drink), we used a negative binomial mixed regression model. All models included a random component capturing the intra-study programme correlation. Statistical procedures were carried with SAS 9.2.

## Results

On average, students were aged 21.5 (std = 3.3), and mainly female (Table 1). Women were slightly younger compared with men (21.4 *vs* 21.8, F = 21.5, p < 0.01) but there was no significant association between gender and study year (*χ*^2^ =1.18, *p* = 0.28). On average students have been attending the University for about 2.8 years (std = 1.7) and were pursuing bachelor degrees. A majority of students were staying on the campus (66.9%) and in a dormitory (64%), with an average of 4.4 room-mates (std = 4.2). A minority of the students (12.3%) was highly involved in traditional student folklore with a score of 2 to 3. Most students pre-party (67%), with an average of 2.3 pre-parties per month (std = 3.3).

**Table 1:** Socio-demographic and drinking patterns, drinking at college study Belgium 2010: descriptive statistics

**Variables (Unit)**	**Mean (std) or %**	**N**
**Drinking behaviour**		
Never drank alcohol in the last year (%)	6.0	6,992
Age when drank for the first time	15.7 (1.8)	6,643
Drinking frequency (occasions per month)	7.0 (6.6)	6,992
Number of drinks (per day)	1.7 (2.1)	6,992
Frequency of abusive drinking (occasions per month)	2.8 (4.4)	6,992
Heavy drinking - WHO norm (%)	74.6	6,992
Non- or moderate drinker		
Heavy drinker	25.4	
**Socio-demographic**		
Gender (%)	42.7	6,906
Men		
Women	57.3	
Age (years)	21.5 (3.3)	6,906
**College environment**		
Programme (%)		6,906
Bachelor	60.9	
Master	39.1	
Time attending the University (years)	2.8 (1.7)	6,992
Living arrangements	64.0	6,992
Dormitory		
Living with parents	36.0	
Living on the campus	66.9	6,906
Yes		
No	33.1	
Room-mates (number)	4.4 (4.2)	
**Social involvement**		
Student representative	6.1	6,906
Yes		
No	93.9	
Participation in folklore (score)	12.3	6,992
High (2-3)		
Medium (1)	11.3	
None (0)	76.4	
Pre-partying (occasions per month)	2.3 (3.3)	6,992
**Normative and experiential factors**		
Permissiveness (score 0-16)	5.2 (2.3)	6,992
Drinking of friends (drinks per day)	3.5 (4.4)	6,992
Drinking of same-sex students (drinks per day)	3.9 (4.9)	6,992
Drinking of students in general (drinks per day)	4.2 (4.7)	6,992
Number of positive consequences	5.0 (3.1)	6,992

There were some socio-demographic differences between our sample and the population. Compared with the overall University population, our sample had a higher frequency of females (sample: 57.3%; population: 54.6%), was younger (21.5 y vs 21.9 y), and had a higher proportion of undergraduates (62% vs 59%). Overall, these differences were small and do not indicate a systematic tendency towards more or less frequent drinking: women generally drink less than men and undergraduates generally drink more than postgraduates.

On average, students had their first drink at the age of 15.7 (std = 1.8), while only a small percentage had never drink alcohol (6%). In Belgium, the legal drinking age is 16. On average, a student drank seven times a month (std = 6.6), had 1.7 drinks a day (std = 21), and 2.8 episodes of abusive drinking per month (std = 4.4). Over the last year, the students acknowledged on average 5 positive consequences (std = 3.1). The three most frequent consequences were to “approach a person that I probably wouldn’t have spoken to otherwise” (68%), to “find it easy to engage in a conversation in a situation in which I would usually have stayed quiet” (65%), and to feel “like I had enough energy to stay out all night partying or dancing” (64%).

College students overestimated what a typical student drinks; this overestimation decreased for closer-reference students: 4.2 (std = 4.7) daily drinks for students in general, 3.9 (std = 4.9) for same-sex students, 3.5 (std = 4.4) for friends (to be compared with a self-declared 1.7). College students overestimated their friends’ drinking by 2 drinks a day.

Overall socio-demographic variables played a more important role for abusive drinking and number of drinks than for the frequency of drinking (Table 2, Model 1). Men drank more, more frequently, and drank abusively more often than women. But the gender difference was somewhat lower for frequency of drinking (OR = 1.58) and higher for abusive drinking (OR = 2.29). Older students were less likely to drink and, in particular, less likely to engage in abusive drinking. For each additional year of age, the frequency of abusive drinking decreased by 9% and the frequency of drinking decreased by 2%.

**Table 2:** Effect of socio-demographic factors, college environmental factors, and experiential and normative factors on drinking behaviour, drinking at college study Belgium 2010: odds ratio of the negative binomial regression and 95% confidence interval (n = 6,906)

	**Frequency of drinking**				**No. of drinks**				**Frequency of abusive drinking**			
	**Model 1**		**Model 2**		**Model 1**		**Model 2**		**Model 1**		**Model 2**	
	**OR**	**95% CI**	**OR**	**95% CI**	**OR**	**95% CI**	**OR**	**95% CI**	**OR**	**95% CI**	**OR**	**95% CI**
**Gender**												
Male	1.58	(1.55 - 1.61)	1.34	(1.32 - 1.37)	2.14	(2.05 - 2.23)	1.65	(1.58 - 1.72)	2.29	(2.22 - 2.37)	1.71	(1.65 - 1.77)
Female (ref)	1.00		1.00		1.00	.	1.00	.	1.00		1.00	
**Age (y.)**	0.98	(0.97 - 0.98)	1.00	(1.00 - 1.00)	0.93	(0.92 - 0.94)	0.96	(0.95 - 0.97)	0.91	(0.91 - 0.92)	0.95	(0.94 - 0.95)
**Time attending University (y.)**	1.07	(1.07 - 1.08)	1.04	(1.03 - 1.05)	1.08	(1.07 - 1.10)	1.03	(1.02 - 1.05)	1.11	(1.09 - 1.12)	1.05	(1.04 - 1.06)
**Living on the campus**												
Yes	1.15	(1.11 - 1.19)	1.02	(0.99 - 1.05)	1.34	(1.25 - 1.43)	1.10	(1.03 - 1.17)	1.56	(1.48 - 1.64)	1.21	(1.15 - 1.27)
No (ref)	1.00		1.00		1.00		1.00		1.00		1.00	
**Room-mates (no.)**	1.05	(1.04 - 1.06)	1.02	(1.02 - 1.03)	1.06	(1.05 - 1.07)	1.02	(1.01 - 1.02)	1.06	(1.06 - 1.07)	1.02	(1.01 - 1.02)
**Living arrangements**												
Dormitory	0.92	(0.84 - 1.00)	1.02	(0.94 - 1.10)	0.82	(0.74 - 0.91)	0.94	(0.87 - 1.03)	0.85	(0.80 - 0.90)	1.00	(0.94 - 1.06)
Living with parents (ref)	1.00		1.00		1.00		1.00		1.00		1.00	
**Student representative**												
Yes			1.03	(0.94 - 1.13)			0.97	(0.89 - 1.06)			0.84	(0.78 - 0.89)
No (ref)			1.00				1.00				1.00	
**Involvement in folklore**												
High			1.46	(1.37 - 1.57)			1.82	(1.72 - 1.92)			2.11	(2.04 - 2.19)
Medium			1.16	(1.08 - 1.24)			1.34	(1.26 - 1.42)			1.57	(1.50 - 1.63)
None (ref)			1.00				1.00				1.00	
**Pre-partying (times per month)**			1.06	(1.06 - 1.07)			1.08	(1.07 - 1.08)			1.08	(1.08 - 1.09)
**Permissiveness (score 0-16)**			1.08	(1.07 - 1.09)			1.07	(1.06 - 1.08)			1.06	(1.05 - 1.06)
**Drinking by friends (drinks per day)**			1.02	(1.01 - 1.03)			1.03	(1.02 - 1.03)			1.02	(1.02 - 1.03)
**Drinking by students in general (drinks per day)**			0.99	(0.98 - 0.99)			0.99	(0.99 - 1.00)			0.99	(0.99 - 0.99)
**Positive consequences (no.)**			1.10	(1.09 - 1.10)			1.11	(1.10 - 1.12)			1.10	(1.09 - 1.10)
**Covariance Parameter (study programme)**	0.03	(0.02-0.05)	0.01	(0.01-0.03)	0.05	(0.03-0.11)	0.01	(0.00-0.03)	0.23	(0.15 – 0.38)	0.09	(0.06 – 0.17)
**−2LL**	19001		18805		21694		18907		43029		30601	

Higher exposure to college environmental factors meant, in most cases, more frequent, and more abusive drinking (Table 2, Model 1). These risk factors were, in general, more important for excessive drinking than for frequency of drinking. For each additional year spent at the university, drinking became more frequent and the frequency of abusive drinking increased (OR = 1.11). Compared with not living on the campus, living on the campus meant more frequent and more abusive drinking behaviour (OR = 1.56). The greater the number of room-mates, the higher the risk of frequent and abusive drinking behaviour. Each additional room-mate increased the frequency of abusive drinking by 6%. There was one exception to these college environmental factors: staying in a dormitory was associated with less frequent drinking behaviour. This could be due to the collinearity with living on the campus and the number of room-mates: as 93% of those living in dorms were on the campus, it was difficult to disentangle the campus effect from the dormitory effect. We checked this issue in two ways. First, we ran Model 1 for the number of drinks per day, by excluding the “living on the campus” variable. We found that, indeed, living in a dormitory was associated with an increased number of drinks compared with living with parents (OR =1.12 95% CI: 1.06-1.18). Second, we compared the two campuses, the one in Louvain-la-Neuve, which is mainly a student town and is known to expose students to numerous drinking opportunities, with the one in Brussels, which has a much more mixed population, controlling for all other variables of Model 1. We found that the Brussels campus had a lower risk (OR = 0.68, 95% CI: 0.60-0.76) compared with the Louvain-La-Neuve campus, suggesting that living on the campus is a more potent predictor of frequent abusive drinking than living in a dormitory.

There was a small intra-class correlation linked to the study programme and this was more important for abusive drinking (0.23) than for frequency of drinking (0.03), suggesting a slight programme effect on abusive drinking but not on drinking frequency. We found the Faculty of Engineering and the Faculty of Social Sciences to have a higher number of drinks per day (Engineering mean = 2.2, *p* < 0.001; Social Sciences Mean = 2.1, *p* < 0.001), with a lower number in the faculties of medicine (1.21, p < 0.001) and psychology (mean = 1.17 NS).

Model 2 adds social-involvement, normative, and experiential factors (Table 2). The more a student was involved in traditional student folklore, the more frequent his or her drinking behaviour, even at the intermediate level of involvement. This was particularly obvious for abusive drinking (OR = 2.11), with a somewhat lower risk for drinking frequency (OR = 1.46). More frequent pre-partying was associated with increased drinking: one additional monthly occasion of pre-partying increased abusive drinking by 8%. However, not all university involvement increased drinking frequency. Being elected as a student representative was associated with a lower risk of drinking, particularly of abusive drinking (OR = 0.84).

Drinking was more frequent as the number of positive consequences increased and as drinking norms became more favourable to drinking. The more a student thought his friends were drinking, the more and the more frequently he drank (OR = 1.02). Likewise, the more a student thought his friends were permissive regarding drinking, the higher the risk of all drinking behaviour, particularly for drinking frequency (OR = 1.08). Drinking frequency or quantity increased by at least 10% for each additional positive consequence a college student experienced.

In most cases, controlling for social engagement, normative, and experiential factors led to a reduction in the risk associated with the college environment. The effect of the number of years attending the University on abusive drinking decreased from OR = 1.11 to OR = 1.05, while the effect of the number of room-mates decreased from 1.06 to 1.02. The effect of living arrangements became insignificant or very small.

The model’s robustness was checked by including age at first drink, a factor likely to capture individual vulnerability. In most cases, the ORs were only slightly affected: the effect of time attending the university on abusive drinking decreased from 1.11 (Model 1, without controlling for age at first drink) to 1.109 (Model 1, with control for age at first drink); the effect of living on the campus on abusive drinking frequency decreased from 1.56 to 1.52; the effect of traditional student folklore from 2.11 to 2.09. Pre-partying frequency was not affected by this kind of sensitivity analysis.

## Discussion

### Main findings

This study confirmed that excessive alcohol consumption is common among college students, with an average of 3 episodes of abusive drinking per month. Greater exposure to college environmental factors, such as living on the campus, a longer spell at university meant more frequent drinking. These community risk factors were more pronounced for excessive drinking patterns than for the quantity or frequency of drinking. Time had a double and mixed effect: older students drink less and less excessively than younger students; however, the longer the period a student has spent in the university, the higher his/her risk of drinking. These effects of college environmental factors were partly explained by social-involvement, experiential, and normative expectations: college students drank for the positive consequences, because they over-estimate the drinking of their friends, or because of other normative expectations.

### Consistency with previous studies

The role of living arrangements has been shown in previous American [31], European [10,32], and cross-comparative [3,33] studies in which living with parents, not living on the campus, and not living in fraternity and sorority houses protected against heavy or abusive drinking. We found that living on the campus was a more potent predictor of frequent abusive drinking than living in a dormitory (both in model 1 and model 2). On the surface, this might seem to contradict a previous European review [10]. However, this is in part because of the strong association between living on the campus and living in a dormitory. This is also consistent with the Harvard School of Public Health college alcohol study which found that living off-campus was a stronger and more significant factor than staying in a dormitory [31]. The finding that the dormitory became non-significant in model 2 suggests that social-involvement, experiential, and normative expectations contribute to explain college environmental factors of drinking behaviours.

Yet, our study shows that the college environment influences drinking behaviour in a much more complex way that involves not only where students live but also the kind of living arrangements, participation in traditional student folklore, the duration of college training, and the type of faculty in which the student is studying. In particular, living in a dormitory with a high number of room-mates and being highly involved in traditional student folklore also play a role in the frequency of abusive drinking. There is thus not one college environmental risk factor but several that relate to different aspects of student life. This may explain why living away from home had a slightly greater effect on heavy drinking in the American (OR = 1.72) or in the international comparison study (OR = 1.61) than in ours (OR = 1.57). The role of dormitory size needs, in particular, to be emphasized and could be explained by innovation diffusion. As adolescent social network studies have shown, teenagers who have a denser social network are more likely to drink than those with less dense social networks [34]. The finding on that pre-partying contribution to the relationship between college environmental factors and frequency of abusive drinking supports this hypothesis. As in previous studies [15], pre-partying was revealed to be a common practice contributing to both drinking behaviour and the influence of community factors on drinking behaviour. College students pre-party to ease the discomfort or awkwardness associated with meeting new people. As hypothesized in a qualitative study, the pre-party is a base to build on when you get to a party, a way to bond with friends, and a social lubricant at a later event to help “hook up” with a partner [26].

Our study shows that abusive drinking increased with the period attending the college, whereas it decreased with age. These two opposite effects were of similar magnitude: this may explain why previous studies have found no clear relationship between age and drinking behaviour [10]: it all depends on the time spent in the university. Few studies have controlled for the time spent in college, so that the protective maturing effect of age was confounded by the risk attached to the time spent attending college. One important prospective American study found, moreover, that heavy drinking decreased with age [35], while there is wide evidence of an association between late adolescent drinking behaviour and subsequent drinking into adulthood [8]. Why did older students drink less while, at the same time, more years at the University were associated with more drinking? Firstly, the correlation between age in years and number of years attending the university was not very high (correlation coefficient = 0.33), suggesting that not all students follow the same trajectory. Some start a postgraduate programme later in life, while working part-time. These “older” students generally spend a shorter period at university (2-3 years) and, possibly, have less time for student activities involving alcohol. Secondly, age and time at the University capture different risks linked to drinking alcohol: age may also capture a cohort effect and, in particular, changes in drinking habits: older students may not only adapt their consumption but may also have started drinking later than the younger age group. This is supported by our data, as we found a small but significant positive correlation between age and age at first drink (correlation = 0.22, p <0.001), although, with our cross-sectional design, these correlations must be approached with caution. A third possible explication is that a significant proportion of students had studied outside the University for their first undergraduate degree and where thus not exposed to the campus for as long as those who followed both under- and postgraduate programmes on the same campus. Our study suggests that the maturing effect on heavy drinking is modest and depends on the time spent attending the University, leaving one particular group of college students at risk: those starting university at a younger age and studying there for longer periods. But these results should be approached with caution. Truncation may affect our results, as younger students who failed to graduate because of heavy alcohol consumption are less likely to be observed at a later stage; this makes the comparison between younger and older students problematic: the latter are observed if they haven’t dropped out of the University.

We found that students overestimate other students’ average number of daily drinks. To compare our results with previous studies of self-other comparison in drinking, we computed the Z Fisher transformation correlation between self-reported daily number of drinks and friends’ numbers of drinks. Our Z Fisher correlation was 0.36 (*p* < 0.001), which compares quite well a Z fisher value of 0.29 from a previous meta-analytic integration of 23 studies [29]. The college social environment increases drinking through a combination of social activities and normative and motivational expectations. It puts students at risk of frequent and abusive drinking because students expect positive social consequences, because of social activities such as pre-parties, and because of injunctive and descriptive drinking norms. The role of such social and normative influences, evidenced in previous studies [36,37], may result from two different and complementary processes: social learning, in which drinking behaviour is acquired through social interaction, and social control, which emphasizes the role of social expectations such as norms and peer pressure [38]. We found that college students overestimate other students’ alcohol consumption and this overestimation decreases with social distance: drinking behaviour was more related to the quantities drunk by friends than to the quantities drunk by students overall. Finally, pre-partying and participation in traditional student folklore, both of which provide strong opportunities for social learning, emerged as strong predictors of drinking behaviour. All this suggests that social learning is a key factor that contributes to the effect of the college social environment on drinking behaviour, as found elsewhere [39].

### Limitations

Our cross-sectional study is vulnerable to reversed causality, so the results need to be interpreted with caution. It could be that involvement in student life and drinking behaviour are confounded by unobserved vulnerability. Extraverted individuals are sensitive to positive social rewards and, thus, more likely to engage in socially-motivated drinking, so the relationship between traditional student folklore and drinking behaviour may be biased upwards. Moreover, the dose-response relationship with involvement in traditional student folklore or with the number of room-mates may downplay this risk of confounding without totally removing it. To assess the risk of confounding we replicated the analysis controlling for the age at which the student reported that he or she started drinking, a factor known to predict a heavy alcohol consumption trajectory [40]. Our results suggest that this kind of self-selection risk may slightly affect our conclusions.

The second limitation has to do with the setting, which unlike other campuses in Belgium or abroad, is much less socially mixed, giving the college environmental factors more clout while mitigating other social control effects. Our results, nevertheless, are in line with a cross-comparative study such as the College Alcohol study in the U.S.A. [23] or the European Amsterdam-Antwerp comparison, which showed living arrangements to be a strong predictor of problematic alcohol use [33]. Finally, it could be that our setting provides a pessimistic picture of community factors and is, in that sense, a good model for reflecting on the community risk factors linked to college drinking behaviour.

## Conclusion: relevance for community health promotion

It is foreseen that in the future most young adults will attend university where, our study shows, they will be exposed to frequent and intensive drinking behaviour. That experience will have subsequent and important consequences lasting into adulthood [8]. Colleges need thus to acknowledge their role in this issue and to commit themselves to lower exposure to excessive alcohol consumption. In particular, they need to combine multi-level strategies: individual, group, and organization-level, from a community health promotion perspective. One danger would be a top-down approach of undertaking community actions in ways that do not consider the realities of student life. A first step would be to involve members of the community in identifying realistic objectives, e.g. limiting excessive consumption, and defining targets, e.g. male students involved in traditional and folklore activities in which hazardous alcohol intake peaks. A second step would be to define interventions, e.g. social-norm interventions that could correct gross miss-perceptions and effectively reduce alcohol consumption [41-43]. Third and fourth steps would be to evaluate what has been implemented, to provide feedback in order to improve and extend interventions, which requires sustained funding, and to analyse gaps between national policies and what is locally feasible. More community-based research is needed to face the problem of hazardous alcohol use, which is persistent and pervasive.

## Competing interests

The authors declare that they have no conflicts of interest relevant to the manuscript submitted to BMC Public Health.

## Authors’ contributions

VL conceived the study, carried out the survey, performed the data analysis, and drafted the manuscript. PN participated in the design of the study, carried out the survey, contributed to analysis and helped draft the manuscript. VES contributed to analysis and helped draft the manuscript. WD contributed to analysis and helped draft the manuscript. All authors read and approved the final manuscript.

## Pre-publication history

The pre-publication history for this paper can be accessed here:

http://www.biomedcentral.com/1471-2458/13/615/prepub
